# Community-Associated *Staphylococcus aureus* from Sub-Saharan Africa and Germany: A Cross-Sectional Geographic Correlation Study

**DOI:** 10.1038/s41598-017-00214-8

**Published:** 2017-03-13

**Authors:** Ulla Ruffing, Abraham Alabi, Theckla Kazimoto, Delfino C. Vubil, Ruslan Akulenko, Salim Abdulla, Pedro Alonso, Markus Bischoff, Anja Germann, Martin P. Grobusch, Volkhard Helms, Jonas Hoffmann, Winfried V. Kern, Peter G. Kremsner, Inacio Mandomando, Alexander Mellmann, Georg Peters, Frieder Schaumburg, Sabine Schubert, Lena Strauß, Marcel Tanner, Hagen von Briesen, Laura Wende, Lutz von Müller, Mathias Herrmann

**Affiliations:** 1 Institute of Medical Microbiology and Hygiene, Saarland University, Homburg, Germany; 2 Centre de Recherches Médicales de Lambaréné (CERMEL), Albert Schweitzer Hospital, Lambaréné, Gabon; 3 Ifakara Health Research and Development Centre (IHRDC), Dar es Salaam, Tanzania; 4 Manhiça Health Research Center, Manhiça, Manhiça, Mozambique; 5 Center for Bioinformatics, Saarland University, Saarbrücken, Germany; 6 Department of Public Health, University of Barcelona, Barcelona, Spain; 7 Fraunhofer Institute for Biomedical Engineering, St. Ingbert, Germany; 8 Center of Tropical Medicine and Travel Medicine, Department of Infectious Diseases, Division of Internal Medicine, University of Amsterdam, Amsterdam, The Netherlands; 9 Division of Infectious Diseases and Travel Medicine, University of Freiburg, Freiburg, Germany; 10 Institute of Tropical Medicine, University of Tübingen, Deutsches Zentrum für Infektionsforschung, Tübingen, Germany; 11 Institute of Hygiene, University Hospital Münster, Münster, Germany; 12 Institute of Medical Microbiology, University Hospital Münster, Münster, Germany; 13 Swiss Tropical and Public Health Institute, Basel, Switzerland

## Abstract

Clonal clusters and gene repertoires of *Staphylococcus aureus* are essential to understand disease and are well characterized in industrialized countries but poorly analysed in developing regions. The objective of this study was to compare the molecular-epidemiologic profiles of *S*. *aureus* isolates from Sub-Saharan Africa and Germany. *S*. *aureus* isolates from 600 staphylococcal carriers and 600 patients with community-associated staphylococcal disease were characterized by DNA hybridization, clonal complex (CC) attribution, and principal component (PCA)-based gene repertoire analysis. 73% of all CCs identified representing 77% of the isolates contained in these CCs were predominant in either African or German region. Significant differences between African versus German isolates were found for alleles encoding the accessory gene regulator type, enterotoxins, the Panton-Valentine leukocidin, immune evasion gene cluster, and adhesins. PCA in conjunction with silhouette analysis distinguished nine separable PCA clusters, with five clusters primarily comprising of African and two clusters of German isolates. Significant differences between *S*. *aureus* lineages in Africa and Germany may be a clue to explain the apparent difference in disease between tropical/(so-called) developing and temperate/industrialized regions. In low-resource countries further clinical-epidemiologic research is warranted not only for neglected tropical diseases but also for major bacterial infections.

## Introduction

According to WHO, neglected tropical diseases (NTD) are caused by defined, often rare, pathogens, and multiple research programs aim at investigating the epidemiology, causative pathogens, and treatment of these NTDs. Conversely, this may suggest that even in resource-limited regions, prevalent pathogens such as *Staphylococcus aureus* are not ‘neglected’, and that the epidemiology and microbiology is rather well known or can be inferred from data from developed countries. Literature and practical experience from these regions, however, tell a different story: diagnosis and treatment of infections caused by common pathogens is hampered by a lack of microbiological facilities and a paucity of epidemiologic data^1^.

*S*. *aureus* is a major public health threat and economic burden to health care systems worldwide causing important morbidity and high attributable mortality. Doubtlessly, *S*. *aureus* is a major pathogen also in (so-called) developing, tropical areas such as Sub-Saharan Africa, frequently causing invasive disease (for review: refs 2–4). In developed regions, epidemiologic registries provide data on the clonal structure of the prevalent isolates^5–8^ yielding insight into the association of clonality, gene repertoire, and disease course. In Sub-Saharan Africa, numerous studies exist describing various *S*. *aureus* strain collections with phenotypic and/or genotypic methods^9–20^, yet, they have been collected from retrospective strain collections, lack accompanying clinical data, are not controlled for hospital acquisition of the isolate/disease, and have not been performed strictly comparing the genotype (as clonal complex [CC] attribution and putative virulence gene content). In other words, cross-sectional molecular epidemiologic studies on both methicillin-sensitive and methicillin-resistant *S*. *aureus* are largely lacking.

Hence, the goal of this study was to investigate the hypothesis that prevalent clones of clinically defined, prospectively collected, non-nosocomial, i.e. community-associated, human *S*. *aureus* isolated in a temperate climate/developed region (Germany) differ with respect to their genetic repertoire and clonal lineage when compared to clones isolated in a tropical/developing region (Sub-Saharan Africa).

## Results

Healthy participants’ and patients’ characteristics are summarized in Tables 1 and 2, respectively. The median age of asymptomatic carriers (volunteers) was 18 (0–61) years and 23 (0–89) years in the African and German study sites, respectively. Patients in Africa had a median age (range) of 3 (0–71) years, in Germany 53 (0–98) years. German patients had a higher rate of previous hospital care, or overall healthcare. German patients more frequently showed risk factors for invasive *S*. *aureus* infection (as reflected by elevated rates of Charlson comorbidity score), and in Germany a larger proportion of clinical isolates was obtained from blood cultures when compared to clinical isolates from Africa. African patients had a higher rate of skin and soft tissue infections, while deep invasive infections of the bone/joint, or respiratory tract were more frequently reported among German patients. Patients with a history of HIV infection were only found in the African group.

**Table 1:** Characteristics of healthy *Staphylococcus aureus* carriers.

	Total	Africa (n = 300)	Germany (n = 300)	p value
Median age in years (range)	22 (0–89)	18 (0–61)	23 (0–89)	ns
Male sex, n (%)	280 (47%)	132 (44%)	148 (49%)	ns
History of hospital admission 6 months until 4 weeks prior to sampling, n (%)	14 (2%)	1 (<1%)	13 (4%)	<0.01
Known HIV infection, n (%)	15 (3%)	15 (5%)	0	<0.001
History of AIDS, n (%)	7 (1%)	7 (2%)	0	ns
History of peripheral vascular disease, n (%)	6 (1%)	0	6 (2%)	ns
History of connective tissue disease, n (%)	8 (1%)	0	8 (3%)	ns
Known diabetes, n (%)	8 (1%)	0	8 (3%)	ns

**Table 2:** Characteristics of patients with *Staphylocococus aureus* infection from Africa and Germany.

		Total	Africa (n = 300)	Germany (n = 300)	p value
Median age in years (range)		29 (0–98)	3 (0–71)	53 (0–98)	<0.001
Male gender, n (%)		348 (58%)	160 (53%)	188 (63%)	ns
History of hospital admission 6 months until 4 weeks prior to sampling, n (%)^a^		157 (26%)	29 (10%)	128 (43%)	<0.001
Healthcare contact last 4 weeks, n (%)^a^		129 (22%)	36 (12%)	93 (31%)	<0.001
Continuous residency in a nursing home before sampling, n (%)		4 (<1%)	2 (<1%)	2 (<1%)	ns
Antibiotic treatment in the last 4 weeks, n (%)^a^		133 (22%)	55 (18%)	78 (26%)	ns
Tuberculosis in the last 6 months, n (%)^a^		4 (<1%)	4 (1%)	0	ns
Antituberculous drugs in the last 4 weeks, n (%)^a^		1 (<1%)	1 (<1%)	0	ns
Risk factors for staphylococcal disease	History of intravenous drug abuse, n (%)	7 (1%)	0	7 (2%)	ns
	Intravenous catheter in place, n (%)	23 (4%)	0	23 (8%)	<0.001
	Other intravascular device in place, n (%)	28 (5%)	0	28 (9%)	<0.001
	Non-vascular foreign body/device in place, n (%)	73 (12%)	3 (1%)	70 (23%)	<0.001
	Known HIV infection, n (%)	26 (4%)	26 (9%)	0	<0.001
Charlson comorbidity score, median (range)		0 (0–12)	0 (0–0)	1 (0–12)	ns
Severity of chronic underlying disorder(s) (McCabe & Jackson classification)	Rapidly fatal underlying disease, n (%)	30 (5%)	19 (6%)	11 (4%)	ns
	Ultimately fatal disease [<5 years], n (%)	58 (10%)	17 (6%)	41 (14%)	ns
	Non-fatal underlying disease, n (%)	132 (22%)	22 (7%)	110 (37%)	<0.001
Type of infection	Bloodstream infection, n (%)	79 (13%)	29 (10%)	50 (17%)	ns
	Non-bacteremic infection, n (%)	521 (87%)	271 (90%)	250 (83%)	ns
Acute clinical presentation	Severe sepsis, n (%)	38 (6%)	18 (6%)	20 (7%)	ns
	Septic shock, n (%)	7 (1%)	2 (<1%)	5 (2%)	ns
Clinical site(s) of infection	Bloodstream infection, n (%)	79 (13%)	29 (10%)	50 (17%)	0.02
	Superficial skin infection, n (%)	348 (58%)	191 (64%)	157 (52%)	ns
	Deep skin abscess, n (%)	121 (20%)	82 (27%)	39 (13%)	0.002
	Bone, n (%)	22 (4%)	0	22 (7%)	<0.001
	Joint, n (%)	20 (3%)	3 (1%)	17 (6%)	ns
	Muscle, n (%)	11 (2%)	4 (1%)	7 (2%)	ns
	Fascia, n (%)	2 (<1%)	1 (<1%)	1 (<1%)	ns
	Respiratory tract, n (%)	25 (4%)	6 (2%)	19 (6%)	ns
	Heart, n (%)	5 (1%)	0	5 (2%)	ns
	Central nervous system, n (%)	1 (<1%)	1 (<1%)	0	ns
	Urinary tract, n (%)	7 (1%)	0	7 (2%)	ns
	Other, n (%)	87 (15%)	20 (7%)	67 (22%)	<0.001

^a^Data were reported as recalled by the participant. If possible, patient’s healthcare files or demographic surveillance systems (i.e. Mozambique) were used to confirm these data.

1,190 isolates of the 1,200 *S*. *aureus* isolates could be assigned to 32 CC and three singleton STs. For seven isolates, the CC could not be deduced because they belonged to new MLSTs not covered by known array profiles. These isolates were either from Africa (n = 4, ST2734, ST2744, ST2370) or Germany (n = 3 ST2733, ST2678, ST2735). Three isolates (1.3%) that were not CC attributable by Iconoclust were attributed to CCs by affinity propagation (based on their MA profiles).

**Figure 1:**
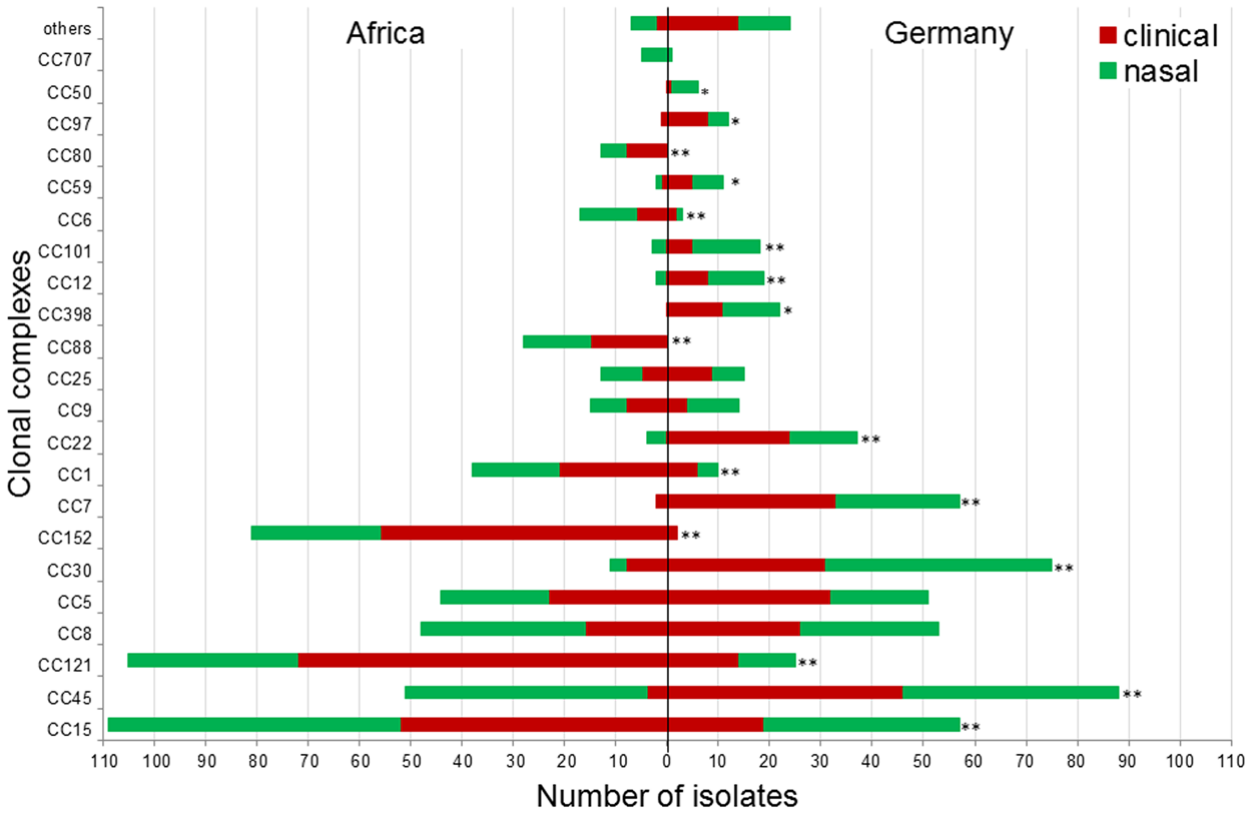
Distribution of the 22 most prevalent clonal complexes (CC) in Africa and Germany among isolates from colonization and infection. CCs of low prevalence (<6 isolates) where grouped together (others). The CCs were sorted in ascending order according to the total number of isolates in the respective CC. The proportions of clinical (red) and nasal (green) isolates in the African and German group are shown. Differences in the distribution of CCs between Africa and Germany were calculated with Fisher’s exact test; *p < 0.05, **p < 0.001.

Figure 1 displays the distribution of CCs of isolates from African and German study sites. Except for four CCs (CC80 and CC88 in Africa, CC50 and CC398 in Germany), all CCs with a number of at least six isolates were found in Africa as well as in Germany. For 17 of the 40 detected CCs and STs, significant geographic distribution differences were found. 16/22 (73%) of the most frequently encountered CCs, and the vast majority of isolates contained within these CCs (896/1168, 77%) were significantly (p < 0.05) predominant either in Africa or in Germany. In the subgroup of clinical isolates, CCs were again significantly (p < 0.05) predominant in Africa or Germany, respectively (with the exception of clusters of low abundance, i.e. CC6 and CC50), while of the CCs contained in the subgroup of commensal isolates, among African isolates only CC88, CC121 and CC152, and among German isolates only CC 7 and CC30 were significantly predominant. In addition, we used already published whole genome sequences of a randomly selected subset of isolates (n = 154) of this study to construct a neighbor-joining tree based on the allelic profiles of 1861 *S*. *aureus* core genome features (cgMLST, Figure S1)^21^. On visual inspection, this analysis also shows that the majority of clusters are based on the geographical region. Clusters of isolates from infection or colonization were not detected.

From the MA repertoire, all genes with a known or presumed regulator, virulence, and/or pathogenicity role were extracted, and compared between isolates from Africa and Germany (Supplementary Table 1). African isolates contain accessory gene regulators *agr* type I through type IV (with apparently some cross-hybridization between *agr*I and *agr*IV as the total number adds up to >100%); in contrast, within German isolates the majority is of *agr*I while *agr*IV was rarely found. Overall, enterotoxin gene recognition was low; yet, *seb* hybridized positively with DNA from African isolates, while *sec*, *sed*, *sel*, and the enterotoxin gene cluster *egc* was preferentially detected in isolates from Germany. A major difference was observed with leukocidins: the genes encoding for the Panton-Valentine leukocidin (PVL) *lukF-PV* and *lukS-PV* were recognized in almost one half of African clinical strains and were virtually absent in German isolates. The *edinA* and *edinB* immune evasion genes encoding the epithelial differentiation inhibitors were more frequently found amidst African isolates as was the gene *isaB* encoding the immunodominant antigen B and the protease gene *splB*. Only fragments and not the full *map* gene encoding the extracellular adhesive protein Eap were detected among African isolates; the gene *sasG* encoding for the biofilm associated surface protein G was more frequently found among African isolates (Table S1).

The majority of resistance genes were equally distributed among isolates from Africa and Germany. In general, methicillin resistance (*mecA*) was low in isolates from Africa (7/300 nasal [2.3%] vs 10/300 clinical [3.3%]) and Germany (2/300 nasal [0.7%] vs 22/300 clinical [7.3%]). Only *blaZ* was more frequently detected in African (560/600, 93%) vs. German isolates (400/600, 67%). Similar results were found for *ermC* (43/600 [7%] vs 91/600 [15%], p < 0.0001) and *tetK* (15/600 [3%] vs 211/600 [35%], p < 0.0001). *merA*, *ermA*, and *tetM* also displayed a significant difference between German and African isolates, yet, at an overall rate of target recognition of less than 10%. These findings correspond well to the phenotypic resistance profiles (Supplementary Table 2); here, striking differences in phenotypic resistance could be observed for tetracycline and trimethoprim-sulfamethoxazole with a larger proportion of resistant isolates in the African population, and clindamycin, with resistance more prevalent among German isolates.

**Figure 2:**
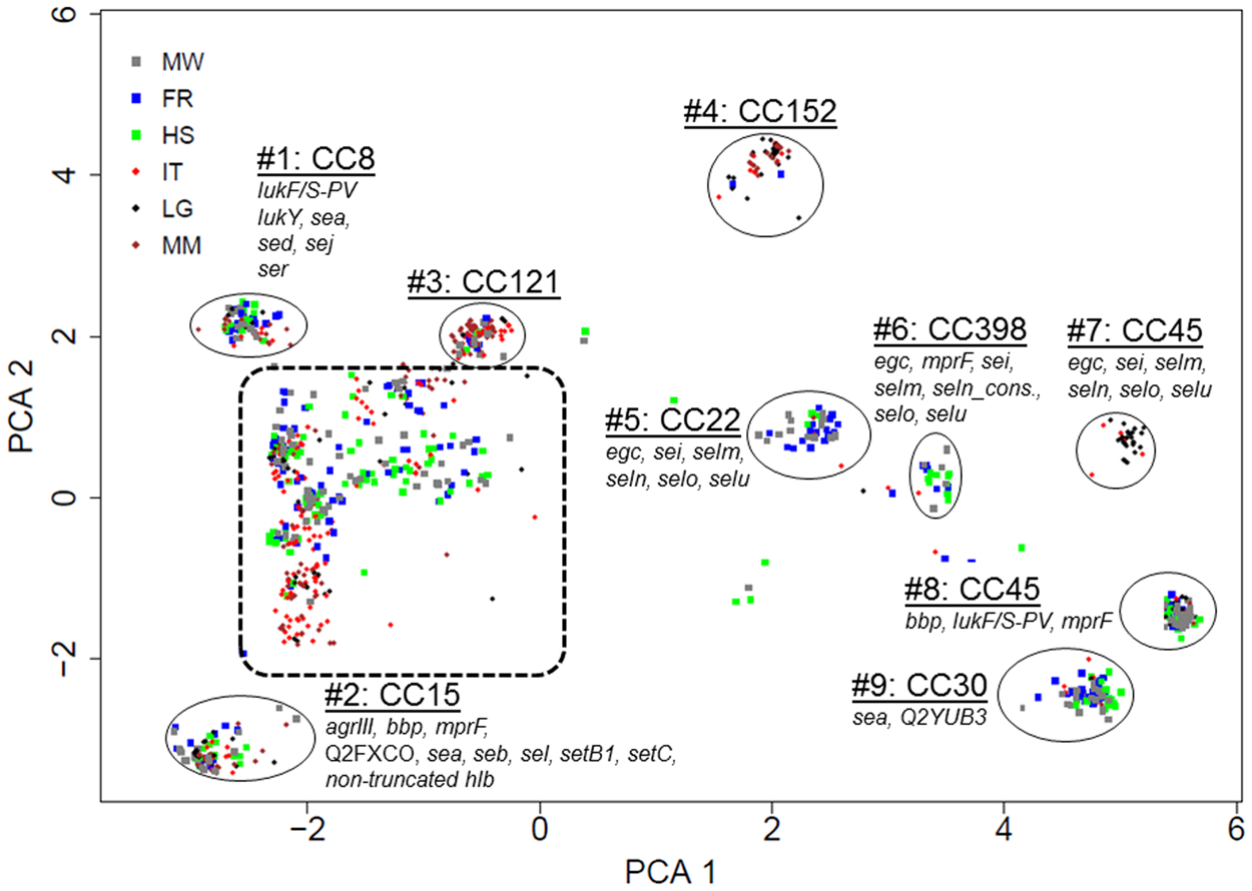
Characteristic genotypic patterns of isolate subgroups detected by DNA microarray. The cluster analysis of 1200 *S*. *aureus* isolates was performed using the principal component analysis (PCA). Each dot represents one isolate. Dots are colour coded according to the study sites in Africa (Ifakara, Tanzania (IT), Lambarene, Gabon (LG), Manhiça, Mozambique (MM)) and Germany (Münster (MW), Freiburg (FR), Homburg (HS)). Major clusters that correspond to multilocus sequence typing clonal complexes (CC) are highlighted. Genes that were significantly (p < 0.01) associated with the respective CC are mentioned. Virulence factors that were significantly associated with ≥4 CCs are not displayed. Isolates encircled with a dashed line belong to CC1, CC5, CC6, CC7, CC9, CC12, CC20, CC25, CC49, CC50, CC59, CC80, CC88, CC97, CC101, CC188, CC395, CC509, CC707, CC913, CC1021, CC1290 or ST580, ST1093, ST2370, ST2733, ST2734, ST2735, ST2744, and ST2678.

The combined PCA/Silhouette analysis allowed to identify nine PCA clusters (labelled #1–9, Fig. 2). Overall, the CC attribution of the isolates corresponded to these PCA clusters, i.e. the isolates confined to a PCA/silhouette cluster could be attributed to a specific CC. Clusters with preferential composition of ‘African’ isolates are primarily found on the left side of the PCA plot (#2 [CC15], #3 [CC121], #4 [CC152]), whereas the clusters on the right side of the plot were preferentially of ‘German’ provenance (#6 [CC398], and #9 [CC30]). CC45 can be separated into two clusters (cluster #7 and cluster #8) of different geographic origin. In addition to these well-defined clusters (#1–9), there are additional clusters of isolates (Fig. 2, dashed line) which are associated with various CCs (i.e. CC1, CC5, CC6, CC7, CC9, CC12, CC20, CC25, CC49, CC50, CC59, CC80, CC88, CC97, CC101, CC188, CC395, CC509, CC707, CC913, CC1021, CC1290) or STs (i.e. ST580, ST1093, ST2370, ST2733, ST2734, ST2735, ST2744, ST2678).

With the Kolmogorov-Smirnoff test we identified the MA hybridization targets, which distinguished the isolates in the respective clusters #1–9 out of all MA hybridization signals for each isolate of the collection; these genes are denoted in Fig. 2.

## Discussion

Here we present a prospective, cross-sectional geographic comparative study on strictly community-associated *S*. *aureus* isolates recovered under controlled, identical conditions in Germany and Sub-Saharan Africa and demonstrate that the cluster repartition among African and German isolates is profoundly inhomogeneous.

Studies from Europe revealed CC45, CC5, CC15, CC30, CC8 to be the most frequently encountered clusters^5, 6, 8, 22–24^. The overall smaller studies from Sub-Saharan Africa indicate that CC5, CC15, and CC30 are prevalent^14, 20^, that MSSA-CC8 has been primarily reported from North Africa (whereas MRSA-CC8 has been found in Central and South Africa), and that CC121 was more frequently reported from Sub-Saharan countries compared to Europe^2^. Methicillin-sensitive CC80 has been more frequently reported from North Africa, and may be related to the community-associated MRSA clone ST80 prevalent in Europe^25^. CC88 isolates are also typically methicillin-resistant; because this cluster has almost uniquely been recovered from African regions, it was attributed the acronym ‘African clone’^2^. The PVL positive clonal complex CC152 may also have originated from Africa^14^, expanded through central Europe, then acquired the methicillin resistance^26^. From these literature reports we conclude: first, ‘typical’ *S*. *aureus* clusters such as CC5, CC15, and CC30 appear to be prevalent in both Europe and Africa. Secondly, another set of ‘typical’ clones (such as CC80, CC88, or CC152) is reported from Africa rather than from Europe, yet, these clones do not seem to make up the bulk of isolates recovered in a non-endemic setting. Third, and probably most importantly, clear-cut studies allowing for frequency comparison between European and African clusters are lacking. The underlying mechanism of different population structures of *S*. *aureus* from Africa and Germany is unclear. The conservation of genomic patterns (e.g. gene clusters) and a subsequent clonal expansion could account for these differences. For instance, the ΦSa 2 prophage which carries *lukF-PV* and *lukS-PV* was integrated in the CC80 lineage at few occasions and subsequently clonally expanded in Africa and Europe^25^. Factors that favor the expansion of one clone in one geographic area might be associated with the bacterium itself (e.g. competition between different clones and species). However, host and environmental factors certainly play as well an important role which should be addressed in future studies.

Our cross sectional, comparative study now proves certain CCs of isolates from Africa to be indeed significantly prevalent (CC15, CC121, CC152) or even unique (CC88, CC80) compared to Germany. On the other hand, among isolates from Germany, other CCs are either significantly prevalent (such as CC45, CC30, CC7, CC22) or unique (CC398, CC50). PCA, avoiding multiple comparisons of single target recognition, confirmed this analysis allowing clear separation of the predominant ‘African’ from the ‘German’ clusters.

Does this clonal repartition imbalance contribute to a difference in disease spectrum? In Africa, higher rates of *S*. *aureus-*related pyomyositis are reported, frequently with bone, skin, and soft tissue involvement^15, 27^, at times presenting with multifocal lesions^28^. Moreover, *S*. *aureus* is particularly frequent in skin and soft tissue infections in Africa^2^. Molecular epidemiologic studies (from US and Europe) describe CC15 (in our study, ‘African’), CC30 (‘German’), and CC5, CC8, CC25 (in our study, ‘balanced’) as associated with invasive disease^7, 23, 29^, yet, they do not provide clear clues towards as to a different disease presentation as a function of predominant CCs in tropical/temperate geographic areas. Our study now provides such indication as the two CCs in our study significantly linked with clinical (as opposed to commensal) origin were the ‘African’ clones CC121 and CC152 (while the two CCs associated with nasal provenience were the ‘German’ clones CC45 and CC101).

In addition to the clonal repartition difference between Sub-Saharan Africa and Germany, does the gene repertoire composition contained in the respective, imbalanced CCs contribute to different disease presentation? In our analysis, *agr*IV was identified with an over-representation in African and *agr*I in German isolates, respectively, consistent with previous studies demonstrating *agr*IV to be prevalent in African CC121^30^. Moreover, the previously reported difference in the positivity rate for *lukF-PV and lukS-PV* was clearly confirmed also in this study^14, 15, 31^. The enterotoxin gene *seb* was also found to be predominant in African isolates, in line with results from studies performed in isolates from remote pygmy populations^31^, particularly among isolates of CC121. Of note is the difference in recognition of *isaB* target encoding a gene only expressed *in vivo* ^32^ inhibiting autophagic flux, thus allowing *S*. *aureus* to evade host degradation^33^. The proteases are also considered of importance to virulence^34^, and *splB* was significantly more often detected in African isolates (while *splE* was predominant in German isolates). Among adhesion factors, significant differences were found for *map*, the gene conferring extracellular adherence protein (Eap) expression^35^ and for the surface protein gene *sasG*. For *map*/*eap* this difference was largely attributable to a lacking recognition of the *eap* variant in isolates of CC152 (an African isolate whose genome did also fail to hybridize with the *sdrC* target) while for *sasG* the difference was mainly attributable to CC121. These observations allow to conclude that not only the clonal attribution but also certain regulatory, pathogenicity and virulence genes are differently distributed when comparing African and German *S*. *aureus* isolates obtained from patients with community associated infection.

The MRSA prevalence in our study was very low (nasal isolates: 2%, clinical isolates: 3%) compared to many other studies from Sub-Saharan Africa (23–55%)^36^. However, these studies should be interpreted with caution as, in contrast to our study, species of *S*. *aureus* and methicillin resistance were not confirmed. It is therefore likely that methicillin resistance is over reported in these studies.

The low rates of methicillin resistance could be also the result of strict exclusion of nosocomial, hospital-associated cases of infection. In accordance to the phenotypic data of many African studies showing a high resistance to penicillin^30^ and tetracycline (21.8–92%)^37^, we found a significant predominance of the beta lactamase operon and of the tetracycline resistance determinants *tetK* and *tetM* in the African isolates. Moreover, the erythromycin resistance genes *ermA* and *ermC* were more frequently found in German and African isolates, respectively (in line with a recent study^38^). In part, these findings were also confirmed by the phenotypic resistance profile demonstrating significant differences in susceptibility to tetracycline (but not to erythromycin).

This study has a number of limitations. First, the discrepancy in population age and comorbidities between the German and African cohort potentially biases the ‘true’ distribution of clones and genes between isolates from the different geographic regions (although application of a multiple linear regression model for the detection rate of Panton-Valentine leucocidin genes failed to provide evidence that age acts as a confounding variable [not shown]). In line, the imbalance in the type of infection (as shown in Table 2) between patients from Germany and Africa may also be a confounder with respect to the CC and virulence gene profile. *Ex ante* we deliberately did not attempt to match patients from Germany and Africa for age, comorbidity profiles, or type of clinical disease; instead, it was our goal to compare the patient characteristics and *S*. *aureus* isolates of a typical patient population presenting for primary medical care at German and African Medical Centers, and to avoid a potential bias incurred by imbalanced strata sizes. Secondly, the MA technique does not allow to distinguish between allelic variants not recognized by hybridization, and complete absence of alleles or genes (this issue has been investigated recently by our group comparing whole genome sequencing (WGS) and MA of exemplar isolates demonstrating that both techniques are highly but not fully reliable with respect to the gene/allele identification [with 1.7% WGS errors and 1.8% MA errors]^39^). Furthermore, the amount of gene transcripts or gene products was not assayed; thus, no correlation between transcript levels and geographic isolate provenience can be inferred. Thirdly, it was not possible to quality control the reliability of clinical case ascertainment beyond the instruction of the clinical personnel on following the written detailed instruction provided together with the structured questionnaires, and attribution of clinical characteristics may therefore lack scrutiny. Fourthly, we did not engage additional study sites from Europe; therefore, our comparison is limited to German isolates. However, in contrast to MRSA, MSSA have a similar population structure across Europe^5^. As the majority of our isolates were MSSA, results from Germany could be used as a surrogate for Europe.

In conclusion, prospectively collected, community-associated *S*. *aureus* isolates obtained from asymptomatic carriers and patients demonstrate profound and significant differences between Germany and various Sub-Saharan African regions, both with respect of clonal cluster attribution and gene repertoire, and for many genes the difference between the cohorts appears to be even more pronounced when only clinical isolates from both regions are analyzed. Thus, based on the overall clonal attribution and allele repertoire, our data provide first clues to explain the purported difference in clinical presentation and course of diseases caused by *Staphylococcus aureus*, a pathogen of major significance both in developing and developed regions.

## Methods

### Study design and participants

This is a cross sectional, geographical correlation study. Wherever applicable, described definitions and items on molecular epidemiology for infectious diseases study designs were applied^40^. Between years 2010 and 2012, a total of 1200 community-associated isolates was collected in three African (Lambaréné, Gabon; Bagamoyo, Tanzania; Manhiça, Mozambique) and three German study sites (Homburg, Freiburg, Münster). Every study site collected 100 non-duplicate isolates of healthy asymptomatic carriers. Exclusion criteria were (i) hospitalization within the past four weeks, (ii) antibacterial treatment within the past four weeks, and (iii) antituberculous treatment in the past four weeks. In addition, 100 clinical non-duplicate isolates were collected from human infection at each study site. The inclusion criteria were (i) clinical suspicion of infection by the treating physician, and (ii) community-onset of disease (outpatient clinic, or <48 h after admission). Clinical data were systematically recorded, electronically transmitted to the Freiburg study site, and checked for data consistency.

Ethical approval was obtained from the Ministry of Health and Social Welfare of Tanzania (A 81–2009), Institutional Ethics Committee of the Medical Research Unit of the International Foundation of the Albert Schweitzer Hospital (CERIL 15/09), National Committee of Bioethics for the Health System Mozambique (325/CNBS/12), Ethics Committee of the University of Münster (2009-227-b-S), Ethics Committee of Freiburg (248/09_120491) and the Ethics Committee of the Chamber of Physicians of Saarland (19/09). A written informed consent was obtained from all study subjects or their legal guardians. All experiments were performed in accordance with relevant guidelines and regulations.

### Isolate collection and microbiological methods

Nasal swabs from asymptomatic carriers and appropriate specimens from infection sites were collected, and species identification performed by standard methods and confirmed at the Homburg site by MALDI-TOF (BRUKER Daltonics GmbH, Bremen, Germany). Antimicrobial susceptibility testing was performed at the various study centers using standard techniques (Clinical and Laboratory Standards Institute, M100).

All isolates were transferred into storage tubes, and shipped on dried ice to a central sample repository (Fraunhofer IBMT, Sulzbach, Germany) for long-term storage at −140 °C.

### DNA microarray-based genotyping and MLST

All isolates were genotyped using the IdentiBAC® DNA microarray (MA, Alere Technologies GmbH, Jena, Germany). DNA extraction (Qiagen, Hilden, Germany) and hybridization were performed according to the manufacturer’s instructions. Spot signals were analyzed using ArrayMate® reader and corresponding Iconoclust® software (Alere Technologies GmbH, Jena, Germany) attributing specific multilocus sequence typing (MLST) clonal complex (CC) and sequence type (ST) designations. MLST was carried out for samples that were not assigned by the MA^41^.

### CC assignment confirmation and statistics

Correctness of the CC identification by MA was confirmed by WGS of 154 exemplars^39^ defined by affinity propagation^42^. Principal component analysis (PCA) was performed to represent the isolate genotype in a two-dimensional projection. Given a large set of data, PCA identifies a small number of uncorrelated variables (termed principal components) that explain the maximum amount of variance in the data. In particular, the first two variables termed PCA1 and PCA2 describe the largest and second-largest variance in the data (Fig. 2).

Statistical analysis (Kolmogorov-Smirnoff test, Hommel p-value adjustment) was used to determine genotypic differences of isolate clusters defined by PCA. All comparisons were statistically analyzed by Chi-Square adjusted for multiple testing (Hommel p-value adjustment). Chi-Square, multivariate and principal component analysis were performed with the software “R”, version 3.2.0. Silhouette analysis was carried out to determine the number of different isolate clusters in the PCA, and was performed with “R”, version 3.2.2, function silhouette and package “cluster” version 2.0.3 on default parameters.

## Electronic supplementary material

Supplementary Information

## References

[1] Herrmann M (2013). Staphylococcal disease in Africa: another neglected ‘tropical’ disease. Future Microbiol..

[2] Schaumburg F, Alabi AS, Peters G, Becker K (2014). New epidemiology of *Staphylococcus aureus* infection in Africa. Clin. Microbiol. Infect..

[3] Abdulgader SM, Shittu AO, Nicol MP, Kaba M (2015). Molecular epidemiology of Methicillin-resistant *Staphylococcus aureus* in Africa: a systematic review. Front. Microbiol.

[4] Huson MA (2014). Methicillin-resistant *Staphylococcus aureus* as a cause of invasive infections in Central Africa: a case report and review of the literature. Infection.

[5] Grundmann H (2010). Geographic distribution of *Staphylococcus aureus* causing invasive infections in Europe: a molecular-epidemiological analysis. PLoS Medicine.

[6] Feil EJ (2003). How Clonal Is *Staphylococcus aureus*?. J. Bacteriol..

[7] Fowler VG (2007). Potential associations between hematogenous complications and bacterial genotype in *Staphylococcus aureus* infection. J. Infect. Dis..

[8] Wertheim HF (2005). Associations between *Staphylococcus aureus* Genotype, Infection, and In-Hospital Mortality: A Nested Case-Control Study. J. Infect. Dis..

[9] Ako-Nai AK, Ogunniyi AD, Lamikanra A, Torimiro SE (1991). The characterisation of clinical isolates of *Staphylococcus aureus* in Ile-Ife, Nigeria. J. Med. Microbiol..

[10] Ghebremedhin B (2009). Emergence of a community-associated methicillin-resistant *Staphylococcus aureus* strain with a unique resistance profile in Southwest Nigeria. J. Clin. Microbiol..

[11] Shittu A (2012). Characterization of methicillin-susceptible and -resistant staphylococci in the clinical setting: a multicentre study in Nigeria. BMC Infect. Dis..

[12] Anguzu JR, Olila D (2007). Drug sensitivity patterns of bacterial isolates from septic post-operative wounds in a regional referral hospital in Uganda. African Health Sciences.

[13] Ramdani-Bouguessa N (2006). Detection of methicillin-resistant *Staphylococcus aureus* strains resistant to multiple antibiotics and carrying the Panton-Valentine leukocidin genes in an Algiers hospital. Antimicrob. Agents Chemother..

[14] Ruimy R (2008). The carriage population of *Staphylococcus aureus* from Mali is composed of a combination of pandemic clones and the divergent Panton-Valentine leukocidin-positive genotype ST152. J. Bacteriol..

[15] Breurec S (2011). Epidemiology of methicillin-resistant *Staphylococcus aureus* lineages in five major African towns: emergence and spread of atypical clones. Clin. Microbiol. Infect..

[16] Okon KO (2009). Cooccurrence of predominant Panton-Valentine leukocidin-positive sequence type (ST) 152 and multidrug-resistant ST 241 *Staphylococcus aureus* clones in Nigerian hospitals. J. Clin. Microbiol..

[17] Moodley A, Oosthuysen WF, Duse AG, Marais E, South African MSG (2010). Molecular characterization of clinical methicillin-resistant *Staphylococcus aureus* isolates in South Africa. J. Clin. Microbiol..

[18] Egyir B (2014). Molecular epidemiology and antimicrobial susceptibility of clinical *Staphylococcus aureus* from healthcare institutions in Ghana. PloS One.

[19] Egyir B (2014). Insights into nasal carriage of *Staphylococcus aureus* in an urban and a rural community in Ghana. PLoS One.

[20] Conceicao T, Coelho C, Santos Silva I, de Lencastre H, Aires-de-Sousa M (2015). *Staphylococcus aureus* in Portuguese former colonies from Africa and the far East: missing data to help fill the world map. Clin. Microbiol. Infect..

[21] Leopold SR, Goering RV, Witten A, Harmsen D, Mellmann A (2014). Bacterial whole-genome sequencing revisited: portable, scalable, and standardized analysis for typing and detection of virulence and antibiotic resistance genes. J. Clin. Microbiol..

[22] Melles DC (2004). Natural population dynamics and expansion of pathogenic clones of *Staphylococcus aureus*. J. Clin. Invest..

[23] Rasmussen G, Monecke S, Ehricht R, Soderquist B (2013). Prevalence of clonal complexes and virulence genes among commensal and invasive *Staphylococcus aureus* isolates in Sweden. PloS One.

[24] Mehraj J (2014). Methicillin-sensitive and methicillin-resistant *Staphylococcus aureus* nasal carriage in a random sample of non-hospitalized adult population in northern Germany. PLoS One.

[25] Stegger M (2014). Origin and evolution of European community-acquired methicillin-resistant *Staphylococcus aureus*. MBio.

[26] Otter JA, French GL (2010). Molecular epidemiology of community-associated meticillin-resistant *Staphylococcus aureus* in Europe. Lancet Infect. Dis..

[27] Sina H (2013). Variability of antibiotic susceptibility and toxin production of *Staphylococcus aureus* strains isolated from skin, soft tissue, and bone related infections. BMC Microbiol..

[28] Ntusi NB, Khaki A (2011). Primary multifocal pyomyositis due to *Staphylococcus aureus*. QJM.

[29] Rieg S (2013). Microarray-based genotyping and clinical outcomes of *Staphylococcus aureus* bloodstream infection: an exploratory study. PloS One.

[30] Kolawole DO (2013). Characterization of colonizing *Staphylococcus aureus* isolated from surgical wards’ patients in a Nigerian university hospital. PloS One.

[31] Schaumburg F (2011). Population structure of *Staphylococcus aureus* from remote African Babongo Pygmies. PLoS Neglect. Trop. Dis.

[32] Mackey-Lawrence NM, Jefferson KK (2013). Regulation of *Staphylococcus aureus* immunodominant antigen B (IsaB). Microbiol. Res..

[33] Liu PF (2015). IsaB Inhibits Autophagic Flux to Promote Host Transmission of Methicillin-Resistant *Staphylococcus aureus*. J. Invest. Dermatol..

[34] Zdzalik M (2012). Prevalence of genes encoding extracellular proteases in *Staphylococcus aureus* - important targets triggering immune response *in vivo*. FEMS Immunol. Med. Microbiol..

[35] Stapels DAC (2014). *Staphylococcus aureus* secretes a unique class of neutrophil serine protease inhibitors. Proc Natl Acad Sci USA.

[36] Falagas ME, Karageorgopoulos DE, Leptidis J, Korbila IP (2013). MRSA in Africa: filling the global map of antimicrobial resistance. PloS One.

[37] Djoudi F (2013). Panton-Valentine leukocidin positive sequence type 80 methicillin-resistant *Staphylococcus aureus* carrying a staphylococcal cassette chromosome mec type IVc is dominant in neonates and children in an Algiers hospital. New Microbiol..

[38] Phaku, P. *et al*. Unveiling the molecular basis of antimicrobial resistance in *Staphylococcus aureus* from the Democratic Republic of the Congo using whole genome sequencing. *Clin*. *Microbiol*. *Infect*. (2016). 10.1016/j.cmi.2016.04.009 27102139

[39] Strauss, L. *et al*. Detecting *Staphylococcus aureus* Virulence and Resistance Genes - a Comparison of Whole Genome Sequencing and DNA Microarray Technology. *J*. *Clin*. *Microbiol*. (2016). 10.1128/JCM.03022-15 PMC4809937 26818676

[40] Field N (2014). Strengthening the Reporting of Molecular Epidemiology for Infectious Diseases (STROME-ID): an extension of the STROBE statement. Lancet Infect. Dis..

[41] Enright MC, Day NP, Davies CE, Peacock SJ, Spratt BG (2000). Multilocus sequence typing for characterization of methicillin-resistant and methicillin-susceptible clones of *Staphylococcus aureus*. J. Clin. Microbiol..

[42] Frey BJ, Dueck D (2007). Clustering by passing messages between data points. Science.

