# Supplementary material for: Community-Associated Staphylococcus aureus from Sub-Saharan Africa and Germany: A Cross-Sectional Geographic Correlation Study
Source: Sci Rep. 2017 Mar 13;7:154. doi: 10.1038/s41598-017-00214-8 (PMC5428059; doi:10.1038/s41598-017-00214-8)
Supplement: Supplementary file 1 — Supplementary Information [file 41598_2017_214_MOESM1_ESM.pdf]

Supporting Information

Community-Associated *Staphylococcus aureus* from Sub-Saharan Africa and Germany: A Cross-Sectional Geographic Correlation Study

Ulla Ruffing[1], Abraham Alabi[2]*, Theckla Kazimoto[3]*, Delfino C. Vubil[4]*, Ruslan Akulenko[5*], Salim Abdulla[3], Pedro Alonso[4,6], Markus Bischoff[1], Anja Germann[7], Martin P. Grobusch[2,8], Volkhard Helms[5], Jonas Hoffmann[9], Winfried V. Kern[9], Peter G. Kremsner[2,10], Inacio Mandomando [4], Alexander Mellmann[11], Georg Peters[12], Frieder Schaumburg[12], Sabine Schubert[1], Lena Strauß[11], Marcel Tanner[3,13], Hagen von Briesen[7], Laura Wende[1], Lutz von Müller[1], Mathias Herrmann[1#]

[1] Institute of Medical Microbiology and Hygiene, Saarland University, Homburg, Germany, [2] Centre de Recherches Médicales de Lambaréné (CERMEL), Albert Schweitzer Hospital, Lambaréné, Gabon;[3] Ifakara Health Research and Development Centre (IHRDC), Dar es Salaam, Tanzania; [4] Manhiça Health Research Center, Manhiça, Mozambique; [5] Center for Bioinformatics, Saarland University, Saarbrücken, Germany; [6] Department of Public Health, University of Barcelona, Spain; [7] Fraunhofer Institute for Biomedical Engineering, St. Ingbert, Germany; [8] Center of Tropical Medicine and Travel Medicine, Department of Infectious Diseases, Division of Internal Medicine, University of Amsterdam, Amsterdam, The Netherlands; [9] Division of Infectious Diseases and Travel Medicine, University of Freiburg, Germany; [10] Institute of Tropical Medicine, University of Tübingen, Deutsches Zentrum für Infektionsforschung, Tübingen, Germany; [11] Institute of Hygiene, University Hospital Münster, Münster, Germany; [12] Institute of Medical Microbiology, University Hospital Münster, Münster, Germany; [13] Swiss Tropical and Public Health Institute, Basel, Switzerland

*second authors, equal contribution

[#] Corresponding author. Current address: University of Münster Medical Faculty, Domagkstr. 3, 48149 Münster, Germany, Phone: +49-251-8355491, Email: mathias.herrmann@uni-muenster.de

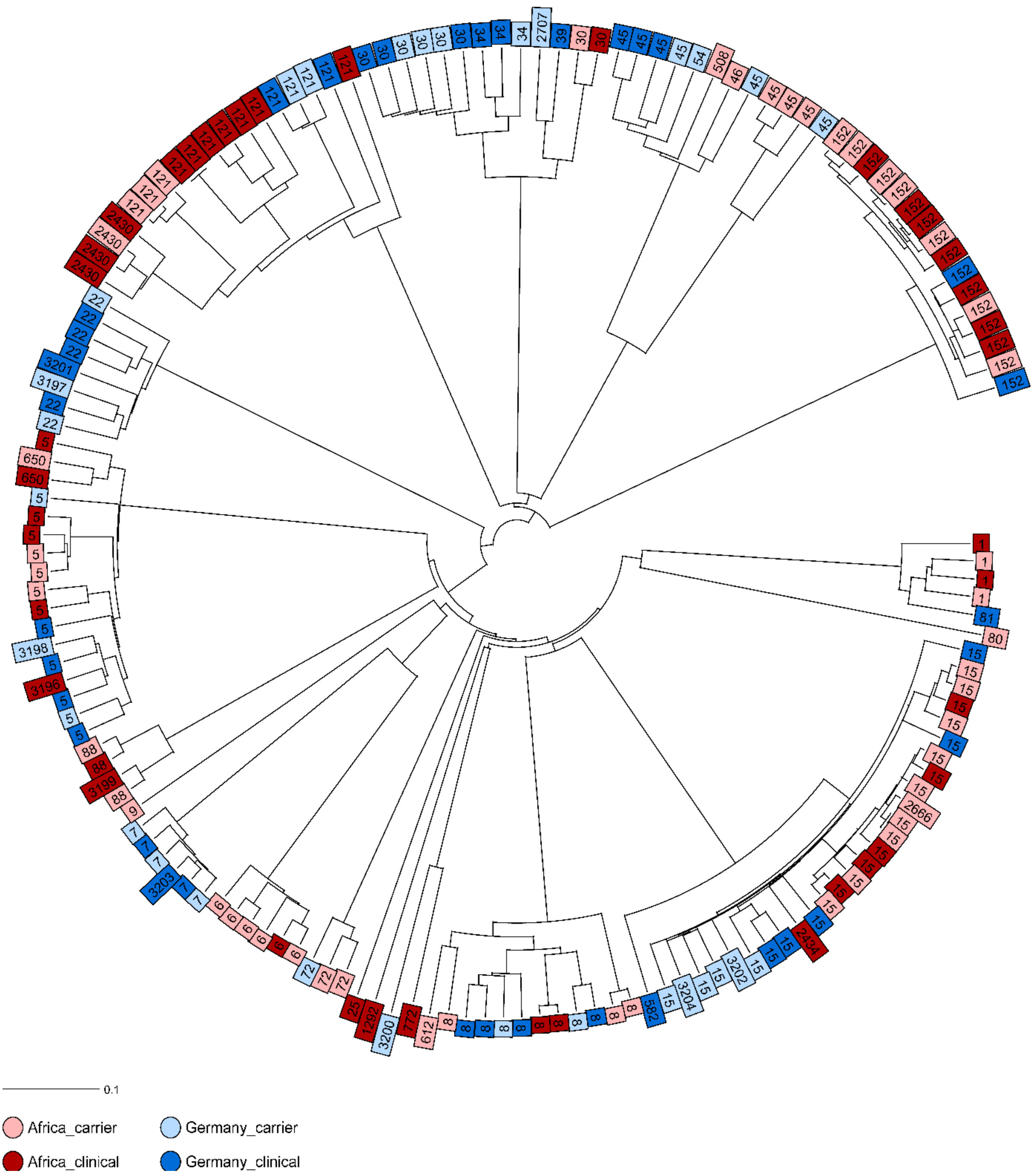

Figure S1: Phylogeny of Staphylococcus aureus from Africa and Germany. *S. aureus* (n=154) from colonization and infection were collected in Africa (red) and Germany (blue). The published whole genome sequencing data were used to construct a neighbor-joining tree (Strauss et al. Clin Microbiol. 2016 Apr;54(4):1008-16). Labels show the multilocus sequence types of each isolate.

Table S1: Microarray target recognition

		All Isolates					Clinical Isolates				
		**German, n (%)**	**African, n (%)**	**OR**	**CI$_{95}$**	**P**	**German, n (%)**	**African, n (%)**	**OR**	**CI$_{95}$**	**P**
Regulatory genes	sarA	599 (100)	600 (100)	n/a			300 (100)	300 (100)	n/a		
	saeS	600 (100)	600 (100)	n/a			300 (100)	300 (100)	n/a		
	vraS	600 (100)	599 (100)	n/a			300 (100)	300 (100)	n/a		
	**agrI total.**	**331 (55)**	**209 (35)**	**2.30**	**1.82-2.91**	**<0.0001**	**179 (60)**	**99 (33)**	**3.00**	**2.15-4.19**	**<0.0001**
	agrII.total	151 (25)	161 (27)	0.92	0.71-1.19	ns	179 (60)	99 (33)	0.83	0.57-1.21	<0.0001
	agrIII.total	84 (14)	93 (16)	0.89	0.65-1.22	ns	68 (23)	78 (26)	0.83	0.57-1.21	ns
	**agrIV.total**	**38 (6)**	**221 (37)**	**0.12**	**0.08-0.17**	**<0.0001**	**36 (12)**	**50 (17)**	**0.10**	**0.06-0.16**	**ns**
Toxins	tst1..consensus	67 (11)	36 (6)	1.96	1.29-3.00	ns	23 (8)	12 (4)	1.9	0.97-4.08	ns
	sea	68 (11)	92 (15)	0.71	0.50-0.99	ns	28 (9)	44 (15)	0.60	0.36-0.99	ns
	**seb**	**45 (8)**	**114 (19)**	**0.35**	**0.24-0.50**	**<0.0001**	**23 (8)**	**72 (24)**	**0.26**	**0.16-0.43**	**<0.0001**
	**sec**	**92 (15)**	**49 (8)**	**2.07**	**1.41-2.94**	**0.02**	**57 (19)**	**19 (6)**	**3.47**	**2.01-5.99**	**0.001**
	**sed**	**52 (9)**	**21 (4)**	**2.62**	**1.56-4.40**	**0.03**	**35 (12)**	**9 (3)**	**4.27**	**2.02-9.05**	**0.01**
	see	1 (0)	0 (0)	n/a			1 (0)	0 (0)	n/a		ns
	seh	26 (4)	34 (6)	0.75	0.45-1.27	ns	12 (4)	18 (6)	0.65	0.31-1.38	ns
	sej	41 (7)	25 (4)	1.69	1.01-1.06	0.01.	27 (9)	10 (3)	2.87	1.37-6.04	ns
	sek	27 (5)	56 (9)	0.46	0.29-0.74	ns	14 (5)	27 (9)	0.50	0.25-0.96	ns
	**sel**	**92 (15)**	**50 (8)**	**1.99**	**1.38-2.87**	**0.03**	**57 (19)**	**20 (7)**	**3.28**	**1.92-5.62**	**0.002**
	**egc total**	**332 (55)**	**253 (42)**	**1.70**	**1.35-2.14**	**0.02**	**173 (58)**	**120 (40)**	**2.04**	**1.48-2.83**	**0.04**
	seq	27 (5)	56 (9)	0.46	0.29-0.74	ns	14 (5)	27 (9)	0.50	0.25-0.96	ns
	ser	37 (6)	20 (3)	1.91	1.09-3.32	ns	24 (8)	8 (3)	3.17	1.40-7.18	ns
	lukF	599 (100)	596 (99)	4.02	0.45-36.07	ns	300 (100)	297 (99)	0.99	0.98-1.00	ns
	lukS	585 (98)	510 (85)	6.88	3.93-12.04	ns	293 (98)	244 (81)	9.61	4.30-21.46	ns
	hlgA	597 (100)	595 (99)	1.67	0.40-7.03	ns	299 (100)	296 (99)	4.04	0.45-36.37	ns
	**lukF.PV**	**15 (3)**	**272 (45)**	**0.03**	**0.02-0.05**	**<0.0001**	**15 (5)**	**187 (62)**	**0.03**	**0.02-0.06**	**<0.0001**
	**lukS.PV**	**15 (3)**	**273 (46)**	**0.03**	**0.02-0.05**	**<0.0001**	**15 (5)**	**188 (63)**	**0.03**	**0.02-0.06**	**<0.0001**
	lukM	1 (0)	0 (0)	n/a			0 (0)	0 (0)	n/a		
	**lukD**	**331 (55)**	**424 (71)**	**0.51**	**0.40-0.65**	**0.004**	**166 (55)**	**215 (72)**	**0.49**	**0.35-0.69**	**ns**
	**lukE**	**326 (54)**	**435 (73)**	**0.45**	**0.36-0.57**	**<0.0001**	**166 (55)**	**220 (73)**	**0.45**	**0.32-0.63**	**0.08**
	hla	597 (100)	598 (100)	0.67	0.11-4.0	ns	297 (99)	300 (100)	n/a		
	hlb	423 (71)	351 (59)	1.70	1.33-2.15	ns	223 (74)	185 (62)	1.8	1.27-2.55	ns
	hld	600 (100)	600 (100)	n/a			300 (100)	300 (100)	n/a		
	etA	24 (4)	39 (7)	0.60	0.36-1.01	ns	9 (3)	19 (6)	0.46	0.20-1.03	ns
	etB	7 (1)	21 (4)	0.33	0.14-0.78	ns	4 (1)	12 (4)	0.32	0.10-1.02	ns
	etD	17 (3)	21 (4)	0.80	0.42-1.54	ns	9 (3)	10 (3)	0.90	0.36-2.24	ns
Immune evasion	sak	466 (78)	477 (80)	0.90	0.68-1.18	ns	243 (81)	246 (82)	0.94	0.62-1.41	ns
	chp	353 (59)	311 (52)	1.33	1.06-1.67	ns	173 (58)	134 (45)	1.69	1.22-2.33	ns
	scn	552 (92)	589 (98)	0.21	0.11-0.42	ns	276 (92)	298 (99)	0.08	0.02-0.33	ns
	**edinA**	**2 (0)**	**26 (4)**	**0.07**	**0.02-0.31**	**0.001**	**2 (1)**	**13 (4)**	**0.15**	**0.03-0.66**	**ns**
	**edinB**	**18 (3)**	**103 (17)**	**0.15**	**0.09-0.25**	**<0.0001**	**10 (3)**	**67 (22)**	**0.12**	**0.06-0.24**	**<0.0001**
	edinC	5 (1)	16 (3)	0.31	0.11-0.84	ns	3 (1)	8 (3)	0.37	0.09-1.40	ns

	ACME total	2 (0)	2 (0)	1.00	0.14-7.12	ns	2 (1)	2 (1)	1.00	0.14-7.15	ns
	**isaB**	**371 (62)**	**529 (88)**	**0.22**	**0.16-0.29**	**<0.0001**	**184 (61)**	**287 (96)**	**0.07**	**0.04-0.13**	**<0.0001**
	mprF	528 (88)	482 (80)	1.80	1.30-2.47	ns	273 (91)	237 (79)	2.69	1.66-4.36	ns
Proteases	aur	553 (92)	585 (98)	0.30	0.17-0.55	ns	278 (91)	296 (99)	0.17	0.06-0.50	ns
	splA	338 (56)	397 (66)	0.66	0.52-0.83	ns	167 (56)	194 (65)	0.69	0.49-0.95	ns
	**splB**	**335 (56)**	**434 (72)**	**0.48**	**0.38-0.62**	**0.001**	**166 (55)**	**222 (74)**	**0.44**	**0.31-0.61**	**ns**
	splE	318 (53)	232 (39)	1.79	1.42-2.25	0.003	144 (48)	107 (36)	1.67	1.20-2.31	ns
	sspA	599 (100)	598 (100)	2.00	0.18-22.15	ns	299 (100)	299 (100)	1.00	0.06-16.06	ns
	sspB	599 (100)	599 (100)	1.00	0.06-16.03	ns	299 (100)	300 (100)	n/a		
	sspP	598 (100)	599 (100)	0.5	0.05-5.52	ns	299 (100)	300 (100)	n/a		
Capsule and Biofilm Genes	cap 5	224 (37)	237 (40)	0.91	0.875-1.393	ns	126 (42)	107 (36)	1.31	0.94-1.81	ns
	cap 8	376 (63)	362 (60)	1.10	0.72-1.14	ns	174 (58)	193 (64)	0.77	0.55-1.06	ns
	icaA	600 (100)	598 (100)	n/a		ns	300 (100)	298 (99)	n/a		
	icaC	597 (100)	514 (86)	33.30	10.47-105.91	ns	298 (99)	243 (81)	34.95	8.45-144.62	ns
	icaD	600 (100)	596 (99)	n/a		ns	300 (100)	298 (99)	n/a		ns
Adhesins	bbp total	543 (91)	575 (96)	0.41	0.26-0.67	ns	271 (90)	286 (95)	0.46	0.24-0.89	ns
	clfA total	600 (100)	600 (100)	n/a		ns	300 (100)	300 (100)	n/a		
	clfB	597 (100)	600 (100)	n/a		ns	299 (100)	300 (100)	n/a		
	cna	284 (47)	321 (54)	0.78	0.62-0.98	ns	148 (49)	170 (57)	0.75	0.54-1.03	ns
	ebh	553 (92)	594 (99)	0.12	0.05-0.28	ns	270 (90)	299 (100)	0.03	0.004-0.22	ns
	ebpS	599 (100)	597 (100)	3.01	0.31-29.02	ns	300 (100)	298 (99)	n/a		
	eno	600 (100)	599 (100)	n/a			300 (100)	299 (100)	n/a		
	fib	372 (62)	448 (75)	0.55	0.43-0.71	ns	184 (61)	231 (77)	0.47	0.33-0.68	ns
	fnbA total	594 (99)	592 (99)	1.34	0.46-3.88	ns	298 (99)	298 (99)	1.00	0.14-1.15	ns
	fnbB total	522 (87)	587 (98)	0.15	0.08-0.27	ns	262 (87)	294 (98)	0.14	0.06-0.34	ns
	**map total**	**582 (97)**	**494 (82)**	**6.94**	**4.15-11.60**	**0.02**	**290 (97)**	**233 (78)**	**8.34**	**4.20-16.57**	**ns**
	**sasG**	**253 (42)**	**370 (62)**	**0.4**	**0.36-0.57**	**<0.0001**	**134 (45)**	**161 (54)**	**0.7**	**0.51-0.96**	**ns**
	sdrC..total.	595 (99)	518 (86)	18.84	7.58-46.82	ns	296 (99)	243 (81)	17.36	6.2-48.52	ns
	sdrD	527 (88)	555 (93)	0.59	0.40-0.87	ns	265 (88)	278 (93)	0.60	0.34-1.05	ns
	vwb..total.	599 (100)	599 (100)	1.00	0.06-16.03	ns	300 (100)	300 (100)	n/a		ns

NB: in bold are targets with a >2-fold or <0.5-fold, statistically different OR of target recognition when comparing German versus African isolates.

Table S2: Rates of *in vitro* antibiotic resistance of *Staphylococcus aureus* from colonization and infection in Africa and Germany

**Source**	**Antimicrobial agent**	**Resistant isolates, % (n)**		**p value**
		**Africa (n=300)**	**Germany (n=300)**	
Colonization	Cefoxitin	2.3% (7)	0.7% (2)	ns
	Tetracycline	35.6% (107)	8% (24)	<0.001
	Erythromycin	20.3% (61)	15.7% (47)	ns
	Clindamycin	4.7% (14)	12.7% (38)	0.005
	Gentamicin	5% (15)	0.3% (1)	0.006
	Trimethoprim-sulfamethoxazole	18.3% (55)	0.3% (1)	<0.001
Infection	Cefoxitin	3.3% (10)	7.3% (22)	ns
	Tetracycline	49.7% (149)	5.7% (17)	<0.001
	Erythromycin	18.7% (56)	19.7% (59)	ns
	Clindamycin	3.7% (11)	14.3% (43)	<0.001
	Gentamicin	1% (3)	2.6% (8)	ns
	Trimethoprim-sulfamethoxazole	19.2% (58)	1.3% (4)	<0.001

NS=not statistically significant
